# Safety of adjuvant gemcitabine plus cisplatin chemotherapy in a patient with bilateral ureteral cancer undergoing hemodialysis

**DOI:** 10.1007/s13691-021-00483-1

**Published:** 2021-04-22

**Authors:** Yumiko Goto, Kent Kanao, Kazuhiro Matsumoto, Ikuo Kobayashi, Keishi Kajikawa, Masafumi Onishi

**Affiliations:** 1 Department of Hospital Pharmacy, Aichi Medical University, 1-1 Yazakokarimata, Nagakute, Aichi 480-1195 Japan; 2 Urology of Uro-Oncology, Saitama Medical University International Medical Center, 1397-1 Yamane, Hidaka, Saitama 350-1298 Japan; 3 Department of Urology, Keio University School of Medicine, 35 Shinanomachi, Shinjuku-ku, Tokyo 160-8582 Japan; 4 Department of Urology, Aichi Medical University School of Medicine, 1-1 Yazakokarimata, Nagakute, Aichi 480-1195 Japan

**Keywords:** Hemodialysis, Bilateral ureteral cancer, Gemcitabine plus cisplatin, Adjuvant chemotherapy

## Abstract

An 80 year old Japanese man with bilateral ureteral cancer underwent laparoscopic bilateral nephroureterectomy and lymph-node dissection. The pathological stage of the left and right ureteral tumors was pT3pN0M0. He received two courses of adjuvant gemcitabine and cisplatin chemotherapy while undergoing hemodialysis. The standard dose of gemcitabine and 50% of the standard dose of cisplatin were administered on the same day. Hemodialysis was started 6 h after gemcitabine administration and 1 h after cisplatin administration. The side effects were evaluated according to the Common Terminology Criteria for Adverse Events v4.0. In the first course, Grade 4 side effects including leukopenia, neutropenia, and thrombocytopenia were observed. He was treated with granulocyte colony-stimulating factor and platelet transfusion. Because the second course was administered without reducing the doses, granulocyte colony-stimulating factor was administered prophylactically, and Grade 4 side effects were reduced to Grade 3. Gemcitabine plus cisplatin chemotherapy can be administered safely in a patient with advanced ureteral cancer undergoing hemodialysis by adequately managing adverse events.

## Introduction

In recent years, the incidence of malignant tumors among patients on maintenance dialysis is high, as the survival rate increases. Gemcitabine plus cisplatin (GC) chemotherapy, and dose-dense (high-dose) methotrexate, vinblastine, doxorubicin, and cisplatin (DDMVAC) chemotherapy, which is possible by the routine use of granulocyte colony-stimulating factor (G-CSF) are first-line chemotherapy options for advanced urothelial carcinoma [1, 2]. The efficiency of GC is known to be almost the same as that of standard MVAC, and the safety and tolerability of GC is higher than that of MVAC [3].

There is no clear evidence on whether preoperative or postoperative chemotherapy for urothelial cancer is better. According to the guidelines for renal pelvic and ureteral cancer treatment established in 2014, postoperative adjuvant chemotherapy may be considered for ≥ pT3 or pN + (recommended grade C1) renal pelvic and ureteral cancer [4], given that their organizations are similar to those of invasive bladder cancer.

The 2016 Renal Impairment Clinical Practice Guidelines suggest that up to 50% of the standard dose of cisplatin (CDDP) may be administered to patients undergoing hemodialysis (HD) because of its nephrotoxicity [5–7]. However, the available evidence for evaluating safety is limited, and to our knowledge, there is no case report of a patient who was administered adjuvant GC chemotherapy after bilateral nephroureterectomy. Herein, we report the case of a patient with bilateral ureteral cancer undergoing HD who was administered adjuvant GC chemotherapy.

## Case report

**Fig. 1:**
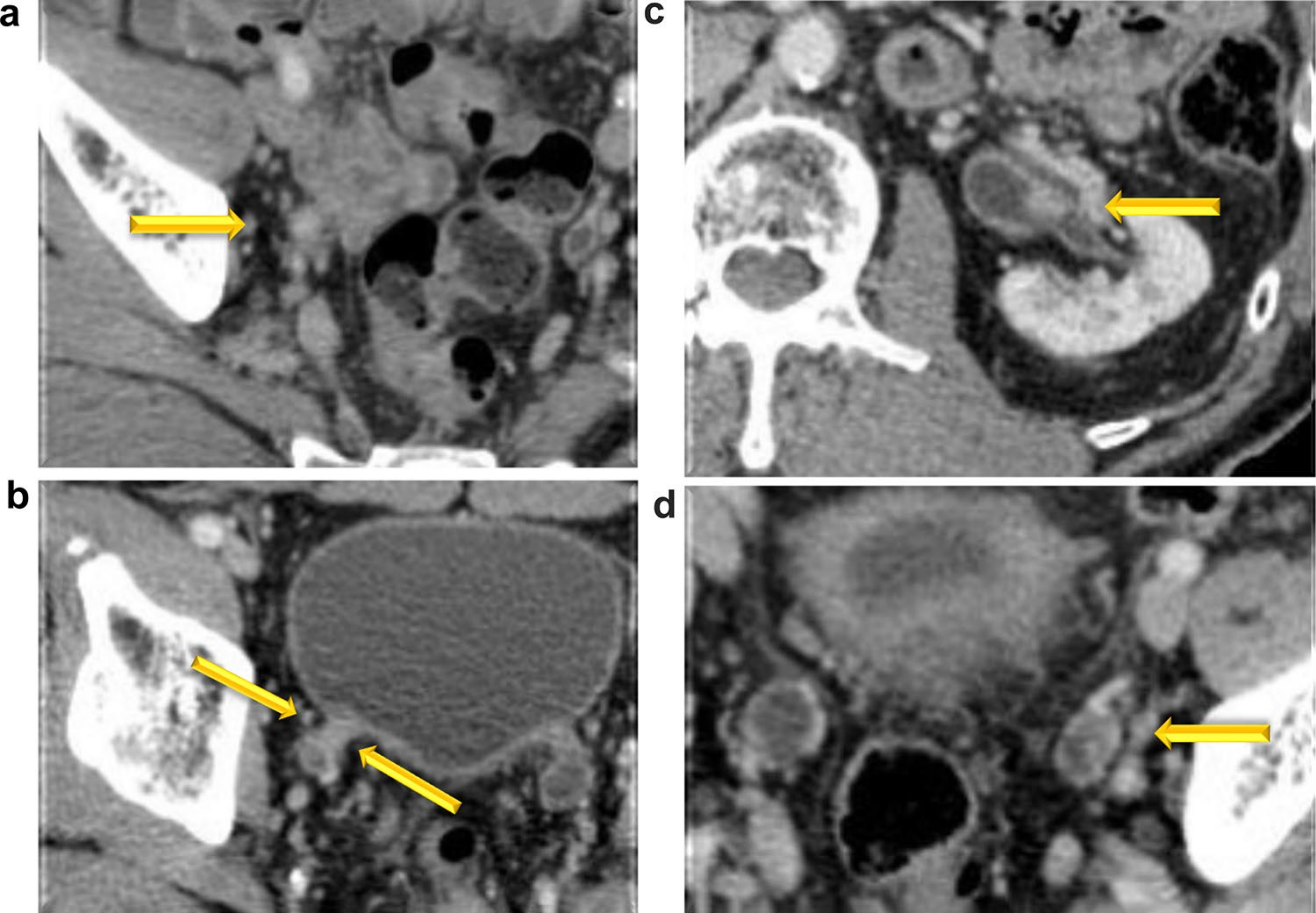
Pre-nephroureterectomy contrast computed tomography showed bilateral ureteral cancer (arrows). Right central ureter (**a**). Right lower ureter (**b**). Left renal pelvis (**c**). Left lower ureter (**d**)

**Fig. 2:**
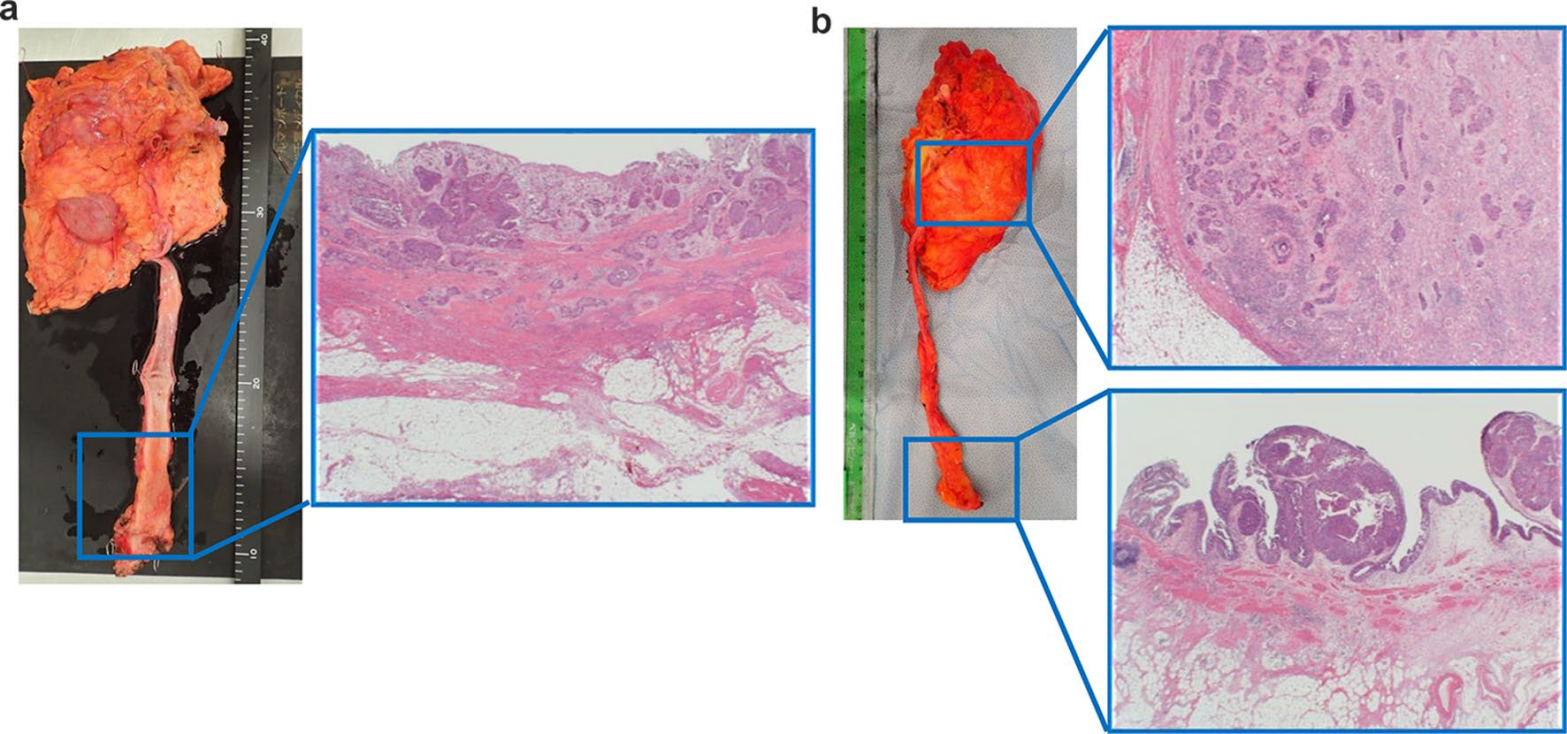
Histopathological findings of the resected tumor of the renal pelvis and ureter. Right (**a**). Invasive urothelial cancer of high-grade pT3 with carcinoma in situ (CIS) observed from the middle to the lower part of the ureter. Left (**b**). Invasive urothelial cancer of high-grade pT3 with CIS was found in the left renal pelvis. CIS was also found in the ureter, which spreaded over almost the entire area

An 80-year-old Japanese man who underwent transurethral ureteral lithotripsy for a right lower ureteral stones at another hospital in 201X suffered a ureteral injury at that time. Post-lithotripsy, he had microscopic hematuria for which he was followed up for 3 months. Following his presentation with gross hematuria for 1 month, he came to our hospital, and bladder cancer was diagnosed. Because of urothelial carcinoma in situ (CIS) after transurethral resection of the bladder tumor (TURBT), Bacille Calmette–Guérin (BCG) was injected into his bladder six times from August 201X + 1. In 201X + 2, a 3 mm papillary tumor recurred in the neck of the bladder [papillary urothelial carcinoma, high-grade (G2 > 3), and non-invasive]. In October 201X + 3, computed tomography findings were suggestive of bilateral ureteral cancer; bilateral laparoscopic nephroureterectomy and lymph-node dissection were performed (Fig. 1). The histopathological examination of the right and left ureteral tumors showed urothelial carcinoma high-grade pT3 with no lymph-node metastasis (Fig. 2). After HD initiation, 2 courses of postoperative GC chemotherapy were administered. Based on the pharmacokinetics of CDDP and gemcitabine (GEM), we used the following regimen. On day 1, he was administered 1000 mg/m^2^ of GEM (100% of normal dose) dissolved in 100 mL of saline intravenously over 30 min, after which 35 mg/m^2^ of CDDP (50% of normal dose) in 250 mL of saline was administered. The total volume of water was 650 mL, and diuretics were not used. HD was started 6 h after GEM infusion, 1 h after CDDP infusion, and it lasted 4 h. HD was conducted 3 times per week. On days 8 and 15, he received 1000 mg/m^2^ of GEM. To prevent nausea, aprepitant, dexamethasone, and palonosetron hydrochloride were administered.

**Fig. 3:**
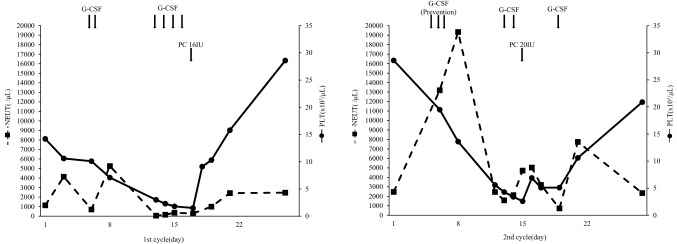
Laboratory data obtained during the patient’s clinical course. Data regarding neutrophils and platelets. Arrows indicate administered granulocyte colony-stimulating factor and platelet concentrates. *NEUT* neutrophils, *PLT* platelet, *G-CSF* granulocyte colony-stimulating factor, *PC* platelet concentrates, *IU* international unit

In the first course, grade 4 leukopenia, neutropenia, and thrombocytopenia occurred. Therefore, GEM was discontinued on day 15 because of bone marrow toxicity. G-CSF and platelet transfusion were administered. Although febrile neutropenia was not observed during the first treatment course, G-CSF was administered proactively on days 5–7 during the second course for prevention of febrile neutropenia. GEM was discontinued on day 15 because of thrombocytopenia; however, neutropenia remained at Grade 3 (Fig. 3). All non-hematological toxicities, such as nausea, fatigue, anorexia, and fever, were Grade 0 throughout all the treatment courses. The patient underwent cystectomy, because multiple recurrent of bladder cancer was observed in the remaining bladder after the two courses of adjuvant GC chemotherapy.

Cystectomy was performed in this patient, because he was diagnosed with high-risk non-muscular invasive bladder cancer with CIS after the first TURBT, and recurrence was diagnosed despite the BCG injections after the first TURBT. The pathological examination of the resected bladder showed invasive urothelial carcinoma (Grade3) with CIS, after BCG and TURBT therapeutic status, pT2, ly0, v0, surgical margin is negative. No recurrence has been observed to date. The patient provided informed consent for the publication of this report and its supporting images. He was managed in accordance with the principles of the Declaration of Helsinki.

## Discussion

We reported a patient who received adjuvant GC chemotherapy safely while undergoing HD. Adjuvant chemotherapy is an accepted standard of care for locally advanced upper urinary tract cancer, based on the result of the PUOT trial [8]; this trial showed that gemcitabine–platinum combination chemotherapy after nephroureterectomy improved disease-free survival significantly. However, given that CDDP is excreted by the kidneys, and it can destroy them, it is used only for patients with good renal functions. When chemotherapy is administered in patients receiving HD, the drug doses and HD schedule are individualized. There is no established treatment regimen for GC in patients receiving HD, and the appropriate dose and timing of each agent for these patients are unclear. There are some reports on urothelial carcinoma treated with CDDP-based chemotherapy in patients receiving HD. Chang et al. [9] carried out a study of 4 patients receiving HD who were treated with a GC regimen based on their experience, but the pharmacokinetics of the drugs were not analyzed. It is best to do careful therapeutic drug monitoring (TDM) and to optimize drug exposure, ensure efficacy, and reduce the risk of side effects [10, 11]. However, a few studies have demonstrated the efficacy and safety of the GC regimen in patients receiving HD by properly monitoring the concentration of each drug. In this case, we had no time to prepare for the TDM of each drug. Therefore, we designed the dose of CDDP and GEM based on pharmacokinetics.

CDDP binds to blood plasma proteins, and bound CDDP exceeds 90% within a few hours after administration [12–14]. The area under the curve of free (unbound) CDDP is closely correlated with cytotoxicity, such as bone marrow toxicity, and other side effects [15, 16]. CDDP has a high protein binding rate, which is expected to rebound immediately after the elimination of free CDDP by HD [13]. Based on previous experience [11, 17], we reduced the dose of CDDP by 50% and performed HD 1 h later.

On the other hand, it was concluded that GEM administration in patients receiving HD is relatively safe [6]. GEM is rapidly metabolized by cytidine deaminase (found in great quantities in the liver, blood, and many tissues) to 2′, 2′-difluorodeoxyuridine (dFdU), which is currently considered an inactive metabolite and which disappears from plasma [14, 18–20].

GEM is rapidly eliminated from plasma even in patients with renal dysfunction. There were no obvious differences in pharmacokinetic parameters, such as the elimination half-life of GEM (t_1/2_), area under the concentration–time curve, and maximum GEM concentration between the patients on HD and those with normal renal functions. On the other hand, the levels of dFdU were constant until HD was initiated, because it was not excreted in urine depending on renal functions. As previously reported, the plasma level of dFdU is reduced by approximately 50% after one HD session [18] and is almost eliminated after 2–3 HD sessions. The same doses of GEM in this study are safe for use in HD and non-HD patients. Meanwhile, dFdU is not toxic, but the clinical effects of its accumulation in the body are unknown. Therefore, it is recommended to perform HD 6–12 h after GEM administration [18, 19].

Following these reasons, we administered a 100% dose of GEM and started HD 6 h after the administration.

After the administration, we did not monitor the blood concentration; frequent blood sampling and observation for side effects were performed. G-CSF and platelet transfusion were administered for neutropenia and thrombocytopenia, respectively.

Since surgery is the basic treatment for urothelial cancer, even a person with a normal renal function may have a unilateral kidney postoperatively, resulting in decreased renal function. Therefore, chemotherapy for patients with urothelial cancer having impaired renal function should be carefully considered. Adjuvant GC chemotherapy was safe for patients with impaired renal function receiving HD after bilateral nephroureterectomy like our patient. These findings may be similar in patients with chronic kidney disease or dialyzed patients. To confirm this, chemotherapy and its side effects should be monitored carefully in these patients in future studies.

In conclusion, adjuvant GC chemotherapy was administered safely in a patient with bilateral ureteral cancer undergoing HD by adequate management of the adverse events.
